# The relationship between metabolic dysfunction-associated fatty liver disease and the incidence rate of extrahepatic cancer

**DOI:** 10.3389/fendo.2023.985858

**Published:** 2023-02-20

**Authors:** Suosu Wei, Yanrong Hao, Xiaofeng Dong, Junzhang Huang, Kai Huang, Yujie Xie, Hongjun Liu, Chunyu Wei, Jinan Xu, Wei Huang, Lingguang Dong, Jianrong Yang

**Affiliations:** ^1^ Department of Scientific Cooperation of Guangxi Academy of Medical Sciences, People’s Hospital of Guangxi Zhuang Autonomous Region, Nanning, Guangxi, China; ^2^ Department of Scientific Research, People’s Hospital of Guangxi Zhuang Autonomous Region, Nanning, China; ^3^ Department of Hepatobiliary, Pancreas and Spleen Surgery, Guangxi Academy of Medical Sciences, People’s Hospital of Guangxi Zhuang Autonomous Region, Nanning, China; ^4^ Department of Breast and Thyroid Surgery, People’s Hospital of Guangxi Zhuang Autonomous Region, Nanning, China

**Keywords:** MAFLD, cancer, metabolic dysfunction, fatty liver disease, incidence rate

## Abstract

**Background:**

The associations between metabolic dysfunction-associated fatty liver disease (MAFLD) and cancer development, especially extrahepatic cancers, are unknown. The aims of the current study were to investigate the cancer incidence rates of MAFLD and analyze the associations between MAFLD and the development of cancers.

**Methods:**

This historical cohort study included participants who underwent ultrasonographic detection of hepatic steatosis at a tertiary hospital in China from January 2013 to October 2021. MAFLD was diagnosed in accordance with *The International Expert Consensus Statement.* Cox proportional hazards regression modeling was used to assess the associations between MAFLD and the development of cancers.

**Results:**

Of the 47,801 participants, 16,093 (33.7%) had MAFLD. During the total follow-up of 175,137 person-years (median 3.3 years), the cancer incidence rate in the MAFLD group was higher than that in the non-MAFLD group [473.5 *vs*. 255.1 per 100,000 person-years; incidence rate ratio 1.86; 95% confidence interval (CI) 1.57–2.19]. After adjustment for age, gender, smoking status, and alcohol status, MAFLD was moderately associated with cancers of the female reproductive system/organs (labium, uterus, cervix, and ovary) [hazard ratio (HR) 2.24; 95% CI 1.09–4.60], thyroid (HR 3.64; 95% CI 1.82–7.30), and bladder (HR 4.19; 95% CI 1.15–15.27) in the total study cohort.

**Conclusion:**

MAFLD was associated with the development of cancers of the female reproductive system/organs (labium, uterus, cervix, and ovary), thyroid, and bladder in the total study cohort.

## Introduction

Non-alcoholic fatty liver disease (NAFLD) is the main cause of chronic liver disease, with a prevalence of 25.0% worldwide (1) and 29.2% in China (1, 2). The prevalence rates of obesity and diabetes are on the rise. As a result, the prevalence of NAFLD will continue to increase, making NAFLD a growing public health problem (3). NAFLD is a risk factor for morbidity and mortality in liver-related diseases and can cause extrahepatic complications such as metabolic syndrome, type 2 diabetes, cardiovascular disease, and chronic kidney disease (4–7). Previous studies indicated that NAFLD can increase the risk of both intrahepatic and extrahepatic cancers, such as stomach, colorectal, lung, thyroid, and breast (8–11).

In 2020, an international panel of liver disease experts proposed changing the name of NAFLD to metabolic dysfunction-associated fatty liver disease (MAFLD) and explained the clinical application of this definition in detail (12). The new definition proposes to remove alcohol consumption or the presence of other liver diseases from consideration in the diagnosis of MAFLD. Patients must be diagnosed with hepatic steatosis in addition to being overweight or obese, have type 2 diabetes, or have two or more metabolic risk factors. Therefore, patients with MAFLD may be diagnosed with other chronic liver diseases (12). The definitions of MAFLD and NAFLD are thus not equivalent. In a cohort study from the UK Biobank, which recruited over 500,000 participants aged 40-69 years, the results showed that MAFLD increased cancer risk by approximately 7.0% overall and 59.0% for liver cancer in particular (13). Another cohort study, which also used data from the UK Biobank to explore the association between MAFLD and 24 specific cancers, showed that MAFLD was significantly associated with 10 of the 24 cancers examined, including cancers of the uterus, gallbladder, liver, kidney, thyroid, esophagus, pancreas, bladder, breast, colorectum, and anus (14). Although studies have shown that MAFLD is associated with cancer risk, individual associations with different sites of occurrence have not been conclusively established. The aims of the current study were to assess the incidences of cancer at different sites in patients with MAFLD and compare them with those in a control population.

## Materials and methods

### Study population and study design

The current historical cohort study included all inpatients diagnosed with MAFLD at the People’s Hospital of Guangxi Zhuang Autonomous Region, China, from January 2013 to October 2021 (registration site http://www.chictr.org.cn/index.aspx; registration number ChiCTR2200058543). Briefly, the study used an advanced medical data management system to manage patients and connected and indexed all diagnosis and treatment records held at the hospital. All the medical information assessed was obtained from the electronic database, including demographic characteristics, medical diagnostic codes, surgical codes, drug prescriptions, and death information. When a patient comes to the clinic, the information is automatically integrated into this system.

In the present study, 251,825 patients undergoing liver ultrasound during hospitalization were selected. Those who had a follow-up time at our hospital of <1 year (*n* = 142,309), lacked information on body mass index (BMI) (*n* = 5,662), or whose cancer events occurred within 1 year of follow-up (*n* = 100) were excluded. Patients with a previous history of discharge diagnosis of liver disease, viral hepatitis, kidney disease, malignant tumor, organ transplantation, or radiation therapy were also excluded (*n* = 38,701). The inclusion and exclusion details are shown in **Supplementary Table 1**. Ultimately, 47,801 patients were analyzed: 16,093 in the MAFLD group and 31,708 in the non-MAFLD group (**Supplementary Figure 1**). The study protocol conformed to the ethical guidelines of the Declaration of Helsinki (6th revision, 2008) and was approved by the Ethics Committees of the People’s Hospital of Guangxi Zhuang Autonomous Region, China. Individual informed consent was not obtained in this study because we analyzed anonymized electronic medical records data as aggregates, with no individual health data available.

### Ascertainment of MAFLD

MAFLD was diagnosed based on abdominal ultrasonography evidence of fatty liver in accordance with the Asia-Pacific Guidelines (15). MAFLD can be diagnosed if fatty liver is diagnosed *via* abdominal ultrasonography and one of the following three conditions exists: 1) overweight or obesity (BMI ≥ 23), 2) diagnosed type 2 diabetes mellitus, or 3) BMI <23 and at least two metabolic risk abnormalities including a) waist circumference ≥90/80 cm, b) blood pressure ≥130/85 mmHg or specific drug treatment, c) plasma triglycerides ≥150 mg/dl (≥1.70 mmol/L) or specific drug treatment, d) plasma HDL-cholesterol <40 mg/dl (<1.0 mmol/L) for men and <50 mg/dl (<1.3 mmol/L) for women or specific drug treatment, e) prediabetes, f) homeostasis model assessment of insulin resistance score ≥2.5 (not included in this study), and (g) plasma high-sensitivity C-reactive protein level >2 mg/L (12) (not included in this study).

### Cancer assessment and covariates

All participants were followed prospectively until death, last medical visit, or December 2022. The International Classification of Diseases–Tenth Revision (ICD-10) codes were used to identify incident cancers (C00–C99). Coding details are shown in **Supplementary Table 2**. In order to minimize spurious diagnoses, all complete medical records of each individual with C00–C99 codes were reviewed by a physician. The covariates assessed in the study included BMI, diabetes mellitus, hypertension, dyslipidemia, and alcohol and smoking status at baseline. BMI was stratified into normal weight (<23) and overweight and obese (≥23). Hypertension was defined as a history of hypertension, the use of antihypertensive medications, or a systolic blood pressure ≥140 mmHg and a diastolic blood pressure ≥90 mmHg. Diabetes was defined as a history of diabetes, taking hypoglycemic drugs, or fasting blood glucose ≥7.0 mmol/L, hBA1c ≥6.5. Dyslipidemia was defined as the use of lipid-lowering agents, LDL-cholesterol >100 mg/dl, or triglycerides >150 mg/dl. All laboratory tests were conducted at the People’s Hospital of Guangxi Zhuang Autonomous Region, China.

### Statistical analysis

Continuous variable baseline data from MAFLD patients and non-MAFLD patients were compared using the Wilcoxon test, and categorical baseline data were compared using the card method. The incidence rates of cancers were calculated by dividing the total number of newly diagnosed cancers by the total number of person-years contributed by people at risk during the follow-up time. Poisson regression modeling was used to estimate the incidence rate ratio (IRR) of cancer progression in the two groups. Cox proportional hazard models were used to estimate hazard ratios (HRs) and 95% confidence intervals (CIs) for the relationships between MAFLD and cancer incidence. Covariate selection was based on a backward selection procedure and other potential confounders identified in the literature (14). In the multivariable analyses, age, gender, smoking status, and alcohol status were adjusted. Stratified analysis by gender was conducted because cancer risks differed between the genders. All reported *p*-values are two-tailed, and *p <*0.05 was considered statistically significant. SPSS 18 software (IBM Corp., Armonk, NY, USA) and R software (version 3.3.2, http://www.r-project.org) were used for the statistical analyses.

## Results

### Baseline characteristics of the study participants

The study included 47,801 participants after the exclusion and inclusion criteria were applied. The prevalence of MAFLD was 33.7% (*n* = 16,093). Participants in the MAFLD group were older, more likely to be male, more likely to smoke, more likely to be diabetic, and more likely to be hypertensive and/or have dyslipidemia. The levels of fasting glucose, total cholesterol, serum alanine aminotransferase, and gamma-glutamyl transferase were higher in the participants with MAFLD. The baseline characteristics of the study participants are summarized in **Table 1**.

**Table 1:** Baseline characteristics of the study participants.

Characteristics	Total (*n* = 47,801)			Male participants (*n* = 19,477)			Female participants (*n* = 28,324)		
	Non-MAFLD (*n* = 31,708)	MAFLD (*n* = 16,093)	*p*-value	Non-MAFLD (*n* = 10,625)	MAFLD (*n* = 8,852)	*p*-value	Non-MAFLD (*n* = 21,083)	MAFLD (*n* = 7,241)	*p*-value
Age (years)	45 (32–62)	60 (50–68)	<0.001	60 (46–69)	58 (48–67)	<0.001	37 (30–55)	62 (52–70)	<0.001
Gender, male (%)	10,625 (33.51%)	8,852 (55.01%)	<0.001			<0.001			<0.001
BMI	22.43 (20.20–24.77)	25.72 (23.94–27.89)	<0.001	22.66 (20.69–24.61)	25.95 (24.22–27.99)	<0.001	22.22 (19.99–24.89)	25.48 (23.62–27.77)	<0.001
Smoking, *n* (%)			<0.001			<0.001			<0.001
Never	28,410 (89.60%)	12,901 (80.17%)	<0.001	7,413 (69.77%)	5,748 (64.93%)	<0.001	20,997 (99.59%)	7,153 (98.78%)	<0.001
Current	1,874 (5.91%)	2,125 (13.20%)	<0.001	1,808 (17.02%)	2,057 (23.24%)	<0.001	66 (0.31%)	68 (0.94%)	<0.001
Past	1,424 (4.49%)	1,067 (6.63%)	<0.001	1,404 (13.21%)	1,047 (11.83%)	<0.001	20 (0.09%)	20 (0.28%)	<0.001
Alcohol, *n* (%)			<0.001			<0.001			<0.001
Never	29,695 (93.65%)	13,932 (86.57%)	<0.001	8,664 (81.54%)	6,758 (76.34%)	<0.001	21,031 (99.75%)	7,174 (99.07%)	<0.001
Current	1,553 (4.90%)	1,696 (10.54%)	<0.001	1,507 (14.18%)	1,665 (18.81%)	<0.001	46 (0.22%)	31 (0.43%)	<0.001
Past	460 (1.45%)	465 (2.89%)	<0.001	454 (4.27%)	429 (4.85%)	<0.001	6 (0.03%)	36 (0.50%)	<0.001
Fasting glucose (mmol/L)	4.50 (4.13–4.98)	5.05 (4.54–6.08)	<0.001	4.61 (4.22–5.14)	5.06 (4.54–6.19)	<0.001	4.45 (4.08–4.89)	5.03 (4.54–5.94)	<0.001
Diabetes, *n* (%)	2,705 (8.53%)	4,479 (27.83%)	<0.001	1,546 (14.55%)	2,508 (28.33%)	<0.001	1,159 (5.50%)	1,971 (27.22%)	<0.001
Hypertension, *n* (%)	7,893 (24.89%)	8,996 (55.90%)	<0.001	4,040 (38.02%)	5,043 (56.97%)	<0.001	3,853 (18.28%)	3,953 (54.59%)	<0.001
Dyslipidemia, *n* (%)	2,736 (8.63%)	5,025 (31.22%)	<0.001	1,384 (13.03%)	2,925 (33.04%)	<0.001	1,352 (6.41%)	2,100 (29.00%)	<0.001
ALT (U/L)	13 (10–19)	20 (14–29)	<0.001	17 (13–25)	23 (16–33)	<0.001	12 (9–16)	17 (13–24)	<0.001
AST (U/L)	19 (16–23)	21 (17–26)	<0.001	21 (17–26)	22 (18–27)	<0.001	18 (15–22)	20 (17–24)	<0.001
GGT (U/L)	17 (12–27)	29 (21–46)	<0.001	26 (18–42)	36 (25–56)	<0.001	14 (11–20)	23 (17–34)	<0.001
TC (mmol/L)	4.74 (3.99–5.65)	4.91 (4.17–5.70)	<0.001	4.49 (3.80–5.27)	4.80 (4.05–5.58)	<0.001	4.92 (4.13–5.87)	5.04 (4.31–5.82)	<0.001
HDL-cholesterol (mmol/L)	1.29 (1.06–1.59)	1.09 (0.93–1.27)	<0.001	1.13 (0.95–1.35)	1.02 (0.88–1.19)	<0.001	1.42 (1.17–1.72)	1.17 (1.01–1.37)	<0.001
LDL-cholesterol (mmol/L)	2.87 (2.33–3.50)	3.06 (2.51–3.64)	<0.001	2.82 (2.28–3.41)	3.02 (2.47–3.59)	<0.001	2.91 (2.36–3.56)	3.12 (2.56–3.70)	<0.001
TG (mmol/L)	1.16 (0.82–1.81)	1.67 (1.21–2.38)	<0.001	1.12 (0.82–1.57)	1.70 (1.21–2.47)	<0.001	1.19 (0.81–2.06)	1.65 (1.21–2.29)	<0.001

Values are expressed as median (Q1–Q3) or number (%).

BMI, body mass index; ALT, alanine aminotransferase; AST, aspartate transaminase; GGT, gamma-glutamyl transferase; TC, total cholesterol; HDL, high-density lipoprotein; LDL, low-density lipoprotein; TG, triglycerides; MAFLD, metabolic dysfunction-associated fatty liver disease.

### Incidence rates of cancer

The follow-up period (median 3.3 years, interquartile range 2.0–5.1 years) included 175,137 person-years of follow-up. Malignancies were newly diagnosed in 291 participants (1.81%) with MAFLD and 290 participants (0.91%) without MAFLD. In the MAFLD group, the overall cancer incidence rate was 473.5 (95% CI 420.6–531.1) per 100,000 person-years, which was significantly higher than that in the non-MAFLD group which was 255.1 (95% CI 226.6–286.2) per 100,000 person-years (IRR 1.86, 95% CI 1.57–2.19) (**Table 2**).

**Table 2:** Cancer incidence rates in participants with and without MAFLD.

Cancer site	Number of cancers	Cancer incidence rates per 100,000 person-years				*p*-value
		All	MAFLD	Non-MAFLD	IRR (95% CI)	
All cancers	581	331.7	473.5	255.1	1.86 (1.57, 2.19)	<0.001
Oral cavity	6	3.4	6.5	1.8	3.70 (0.53, 40.90)	0.105
Pharynx	21	12.0	14.6	10.6	1.39 (0.52, 3.59)	0.456
Esophagus	11	6.3	3.3	7.9	0.41 (0.04, 1.99)	0.240
Stomach	37	21.1	22.8	20.2	1.13 (0.54, 2.28)	0.726
Duodenum, colon, and rectum	104	59.4	79.7	48.4	1.65 (1.10, 2.47)	0.010
Liver	30	17.1	22.8	14.1	1.62 (0.73, 3.54)	0.184
Biliary	8	4.6	8.1	2.6	3.08 (0.60, 19.85)	0.104
Pancreas	12	6.9	11.4	4.4	2.59 (0.71, 10.35)	0.092
Spleen	4	2.3	4.9	0.9	5.55 (0.45, 291.31)	0.095
Laryngeal	6	3.4	8.1	0.9	9.25 (1.03, 437.42)	0.013
Lung	105	60.0	74.8	51.9	1.44 (0.96, 2.16)	0.061
Skin	9	5.1	11.4	1.8	6.47 (1.23, 63.87)	0.007
Breast	37	21.1	30.9	15.8	1.95 (1.02, 3.72)	0.042
Labium, uterus, cervix, and ovary	38	21.7	34.2	15.0	2.28 (1.15, 4.61)	0.009
Prostate	35	20.0	29.3	15.0	1.96 (1.01, 3.80)	0.047
Renal pelvis	13	7.4	14.6	3.5	4.16 (1.16, 18.49)	0.010
Bladder	13	7.4	16.3	2.6	6.17 (1.59, 34.86)	0.002
Brain and central nervous system	10	5.7	11.4	2.6	4.32 (1.12, 16.69)	0.034
Thyroid	41	23.4	35.8	16.7	2.14 (1.11, 4.18)	0.013
Non-Hodgkin’s lymphoma	20	11.4	11.4	11.4	1.00 (0.34, 2.69)	0.993
Leukemia	14	8.0	13.0	5.3	2.47 (0.75, 8.62)	0.084

Overall follow-up duration: 175,137 person-years; non-MAFLD group follow-up duration: 113,678 person-years; MAFLD group follow-up duration: 61,459 person-years.

CI, confidence interval; IRR, incidence rate ratio; MAFLD, metabolic dysfunction-associated fatty liver disease.

In the MAFLD group, the incidence rates of nine specific cancers were significantly higher than those in the non-MAFLD group, including cancers of the duodenum, colon, and rectum (IRR 1.65, 95% CI 1.10–2.47); breast (IRR 1.95, 95% CI 1.02–3.72); female reproductive system/organs (labium, uterus, cervix, and ovary) (IRR 2.28, 95% CI 1.15–4.61); prostate (IRR 1.96, 95% CI 1.01–3.80); thyroid (IRR 2.14, 95% CI 1.11–4.18); renal pelvis (IRR 4.16, 95% CI 1.16–18.49); bladder (IRR 6.17, 95% CI 1.59–34.86); and brain and central nervous system (IRR 4.32, 95% CI 1.12–16.69) (**Table 2**).

With respect to gender, the incidence rate of thyroid cancer in the male participants in the MAFLD group was significantly higher than that in the non-MAFLD group (IRR 3.89, 95% CI 1.07–14.14), but in the female participants, the incidence rates of thyroid cancer did not differ significantly in the two groups. In the female participants, the MAFLD group had significantly higher incidence rates of cancers of the stomach (IRR 3.13, 95% CI 1.05–9.30), lung (IRR 3.01, 95% CI 1.54–5.91), skin (IRR 10.72, 95% CI 1.20–95.88), breast (IRR 2.99, 95% CI 1.56–5.76), and female reproductive system/organs (labium, uterus, cervix, and ovary) (IRR 3.31, 95% CI 1.75–6.27) (**Supplementary Table 3**). The cumulative incidence of all cancers is shown in **Supplementary Figure 2**.

### Association between MAFLD and cancer risk

In the univariate analysis, participants in the MAFLD group had a higher risk of all cancers than those in the non-MAFLD group (HR 1.79, 95% CI 1.52–2.10), and the result was consistent after adjustment for age, gender, smoking status, and alcohol status (HR 1.35, 95% CI 1.15–1.60) (**Table 3**). At different cancer sites, MAFLD was significantly associated with cancers of the female reproductive organ (labium, uterus, cervix, and ovary) (HR 2.24, 95% CI 1.09–4.60), thyroid (HR 3.64, 95% CI 1.82–7.30), and bladder (HR 4.19, 95% CI 1.15–15.27) in the total study cohort. In the subgroup analysis by gender, the results were consistent, with the exception of bladder cancer (**Supplementary Table 4**).

**Table 3:** Association between MAFLD and the development of cancers.

Cancer site	Univariate analysis		Multivariable analysis	
	HR (95% CI)	*p*-value	HR (95% CI)	*p*-value
All cancers	1.79 (1.52, 2.10)	<0.001	1.32 (1.12, 1.55)	0.001
Oral	3.57 (0.65, 19.50)	0.142	2.41 (0.43, 13.60)	0.321
Pharynx	1.32 (0.56, 3.14)	0.525	1.01 (0.42, 2.44)	0.978
Esophagus	0.40 (0.09, 1.85)	0.239	0.30 (0.06, 1.38)	0.122
Stomach	1.08 (0.55, 2.09)	0.825	0.76 (0.39, 1.49)	0.424
Duodenum, colon, and rectum	1.57 (1.06, 2.31)	0.023	1.09 (0.74, 1.61)	0.652
Liver	1.53 (0.75, 3.13)	0.246	1.06 (0.51, 2.17)	0.881
Pancreas	2.54 (0.81, 8.01)	0.112	1.87 (0.59, 5.95)	0.288
Spleen	5.56 (0.58, 53.47)	0.138	3.93 (0.41, 38.02)	0.238
Laryngeal	8.45 (0.99, 72.36)	0.051	5.03 (0.59, 43.11)	0.140
Lung	1.38 (0.94, 2.03)	0.101	0.94 (0.64, 1.38)	0.741
Skin	6.26 (1.30, 30.16)	0.022	4.62 (0.95, 22.38)	0.057
Breast	1.91 (1.00, 3.64)	0.049	1.77 (0.89, 3.53)	0.106
Labium, uterus, cervix, and ovary	2.09 (1.10, 3.99)	0.025	2.24 (1.09, 4.60)	0.029
Prostate	1.88 (0.97, 3.64)	0.063	1.46 (0.75, 2.85)	0.263
Renal pelvis	4.11 (1.27, 13.36)	0.019	2.85 (0.87, 9.32)	0.084
Bladder	5.86 (1.61, 21.31)	0.007	4.19 (1.15, 15.27)	0.030
Brain and central nervous system	4.26 (1.10, 16.49)	0.036	3.73 (0.92, 15.10)	0.065
Thyroid	2.08 (1.13, 3.85)	0.019	3.64 (1.82, 7.30)	0.000
Non-Hodgkin’s lymphoma	0.96 (0.38, 2.42)	0.939	0.71 (0.28, 1.79)	0.466
Leukemia	2.35 (0.81, 6.77)	0.114	1.79 (0.61, 5.26)	0.292

Hazard ratios and p-values represent the MAFLD group compared with the non-MAFLD group, ascertained via Cox proportional hazards regression modeling. Multivariable analyses were adjusted for age, gender, smoking status, and alcohol status.

CI, confidence interval; HR, hazard ratio; MAFLD, metabolic dysfunction-associated fatty liver disease.

## Discussion

In this retrospective cohort study, the relationships between MAFLD and different cancer types were analyzed. MAFLD was associated with higher incidence rates of cancers, particularly extrahepatic-specific cancers of the bladder, thyroid, and female reproductive system/organs (labium, uterus, cervix, and ovary). As MAFLD is a new definition, cohort studies on the relationship between MAFLD and cancer are scarce, with only two cohort studies from the UK Biobank database. The UK Biobank is a large-scale, population-based prospective cohort study that recruited over 0.5 million participants aged 40-69 years in 2006-2010 and combined extensive measurements of baseline data and genotype data with linked national medical records for longitudinal follow-up (13). One study selected 423,252 participants who were diagnosed with MAFLD. During the median follow-up of 8.2 years, compared with participants without MAFLD, those with MAFLD had a multivariate-adjusted HR of 1.07 (95% CI 1.05–1.10) for all cancers and 1.59 (95% CI 1.28, 1.98) for liver cancer (13). The other study included 352,911 participants, with 37.2% diagnosed as MAFLD, and during the median follow-up of 8.2 years, compared with non-MAFLD, MAFLD was significantly associated with 10 of the 24 examined cancers, including corpus uteri (HR = 2.36, 95% CI 1.99–2.80), gallbladder (2.20, 1.14–4.23), liver (1.81, 1.43–2.28), kidney (1.77, 1.49–2.11), thyroid (1.69, 1.20–2.38), esophagus (1.48, 1.25–1.76), pancreas (1.31, 1.10–1.56), bladder (1.26, 1.11–1.43), breast (1.19, 1.11–1.27), and colorectal and anus cancers (1.14, 1.06–1.23) (14). The results of this study are mostly consistent with the two UK Biobank cohort studies, except for liver cancer. In this present study, there was no significant relationship between MAFLD and liver cancer. The discrepancy may be related to differences in study populations in the two studies. The UK Biobank study recruited a representative sample of the general population based on ethnic and sociodemographic data, with an equal proportion of men and women and a balanced population between the ages of 40 and 69 years over a 5-year period (16, 17), whereas our study data were derived from a hospital patient population.

MAFLD is defined differently from NAFLD, which excludes liver-related diseases. The exclusion of other causes such as drug-induced hepatitis, viral hepatitis, alcoholism, and other liver diseases is not a requirement for MAFLD (18). The definition of MAFLD covers the systemic risk associated with fatty liver disease, whereas NAFLD focuses on liver-related factors. Therefore, given the strong association between MAFLD and the established risk factors for these diseases, it is not surprising that MAFLD is associated with an increased risk of both intrahepatic and extrahepatic events (13). Currently, the definition of MAFLD is somewhat controversial in the field of liver disease (19, 20). A meta-analysis showed that MAFLD was not a replacement for NAFLD and that there were significant differences in the prevalence and risk factors between them (21). MAFLD was proposed as an alternative definition in an effort to improve people’s awareness, especially primary care doctors, and to better summarize metabolic dysfunction (22).

In the current study, MAFLD was moderately associated with cancers of the female reproductive system/organs (labium, uterus, cervix, and ovary), bladder, and thyroid in the total study cohort. MAFLD increases the risks of obesity, diabetes, and hypertension, in turn increasing the burden of metabolism-related diseases (21). In the present study, the MAFLD group had a higher median BMI than the non-MAFLD group, and the prevalence rates of diabetes, hypertension, and dyslipidemia were also higher in the MAFLD group. Studies on hypertension and cancer indicate that patients with hypertension have a significantly higher risk of cancer than non-hypertensive patients, particularly with respect to colorectal cancer and breast cancer (23–25). In a two-sample Mendelian randomization study, there were causal detrimental effects of type 2 diabetes on cancer of the uterus, kidney, pancreas, and lung (26). Dyslipidemia and obesity are evidently risk factors for most cancer types (27, 28). This may explain why the cancer incidence rate was significantly higher in the MAFLD group in the current study. The precise pathophysiological mechanisms that link MAFLD and cancers are unknown, and further studies are needed to elucidate these links. Moreover, there may be significant implications for cancer screening and surveillance strategies in MAFLD patients given the growing number of patients with MAFLD.

The strength of the present study is that it is the first large cohort study to explore the association between MAFLD and cancer in western China. However, there are some limitations to this study. First, the participants of this study were derived from a hospital patient population, so the potential selection bias was unavoidable. Second, fatty liver was diagnosed using abdominal ultrasound rather than liver biopsy. Ultrasound has limited sensitivity, and it could result in steatosis with less than 20% of fatty liver individuals that cannot be detected (29, 30). However, due to its invasive nature, liver biopsy is not feasible in large population-based studies. Third, the follow-up time of this study was short (median 3.3. years), leading to outcome events that could not be observed. In addition, the small numbers of some site-specific cancers may have resulted in the instability of the results. Thus, a longer follow-up is required to verify the results. Fourth, the parameters used to assess the diagnosis of MAFLD, such as waist circumference, insulin, oral glucose tolerance test, and hsCRP, are missing in this study. This may have resulted in some MAFLD cases being missed and MAFLD may have misclassification in some participants. So, we will construct a long-term follow-up of a prospective cohort study that has complete baseline data to avoid this bias. Fifth, cancer cases were identified by ICD-10 codes, which may be associated with misclassification or underreporting. Sixth, the pathological severity of hepatic steatosis was not collected in this study. Lastly, metabolic abnormalities may change with the state of the participants, so the metabolic state at baseline may not accurately reflect the true metabolism of the individual.

## Conclusion

In this retrospective cohort study, MAFLD was associated with an increased risk of cancer. The study suggests that multidisciplinary assessment is required and attention should be paid to the development of malignancy in patients with MAFLD.

## Data availability statement

The raw data supporting the conclusions of this article will be made available by the authors, without undue reservation.

## Ethics statement

This study was performed in accordance with the principles outlined in the Declaration of Helsinki and approved by the People’s Hospital of Guangxi Zhuang Autonomous Region Ethics Committee and the Institutional Review Board. Individual informed consent was not obtained in this study because we analyzed anonymized electronic medical records data as aggregates, with no individual health data available.

## Author contributions

SW and JY conceived and designed the study and reviewed the manuscript. All authors contributed to the article and approved the submitted version.
