# Supplementary material for: The relationship between metabolic dysfunction-associated fatty liver disease and the incidence rate of extrahepatic cancer
Source: Front Endocrinol (Lausanne). 2023 Feb 20;14:985858. doi: 10.3389/fendo.2023.985858 (PMC9987419; doi:10.3389/fendo.2023.985858)
Supplement: Supplementary file 1 [file DataSheet_1.docx]

# Supplementary Figure 1. Flow chart of study participants` selection in the medical record system.


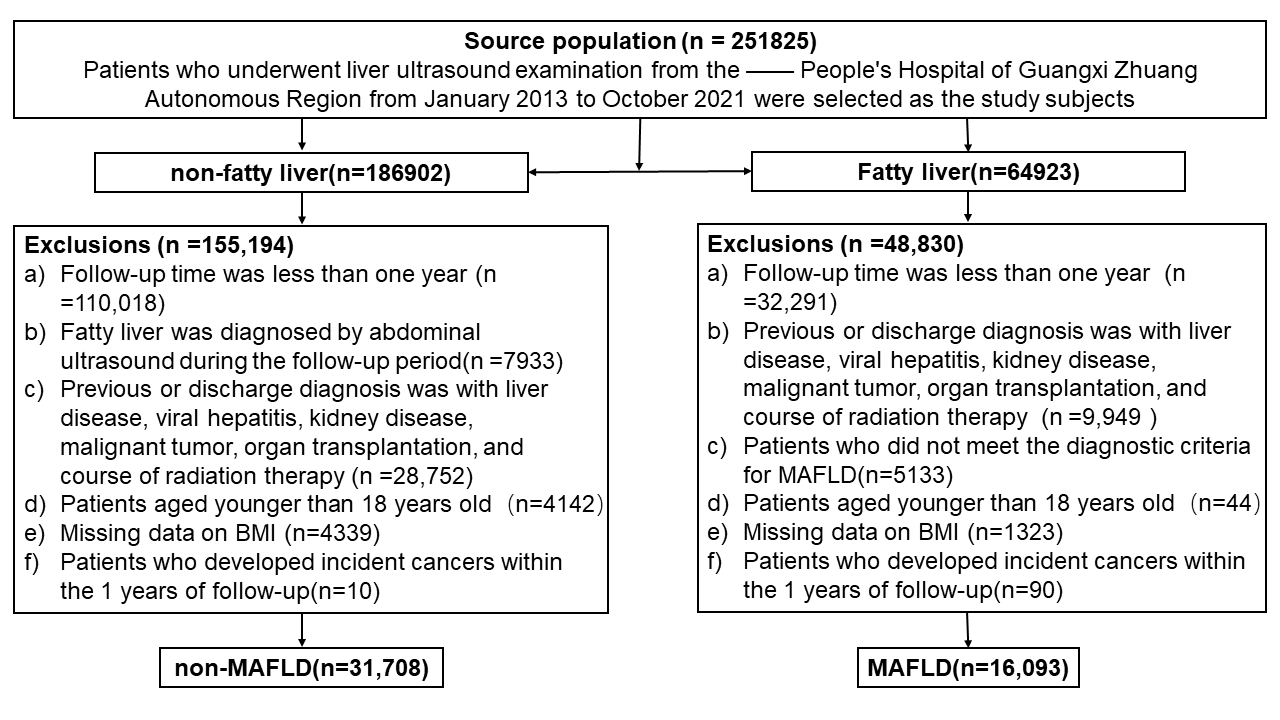


# Supplementary Figure 2. The cumulative incidence of all cancer.


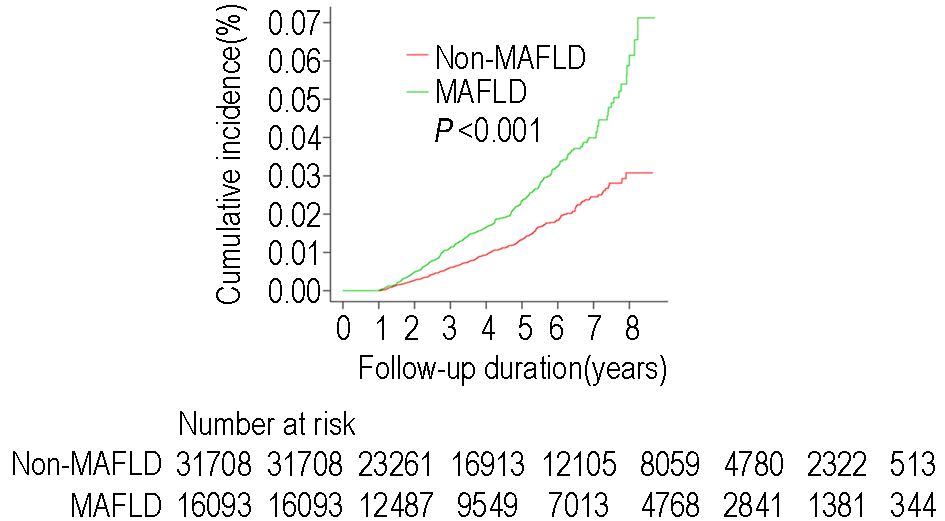


| Supplementary Table 1. Inclusion and exclusion codes used in the algorithm of MAFLD cases identification |
| --- |
| **International Classification of Diseases Codes ( ICD-10)** |
| **Inclusion** |
| K76.00: Fatty liver disease |
| **Exclusion** |
| **1. Hepatic disease** |
| K70.000: alcoholic fatty liver disease |
| K71.000: Toxic liver disease with cholestasis |
| K72.000: Acute and subacute liver failure |
| K73.000: Chronic delayed hepatitis, can not be classified in other places |
| K74.000: Liver fibrosis |
| K75.000: Hepatic abscess |
| K76.000: Fatty liver disease (Fatty liver was diagnosed by abdominal ultrasound during the follow-up period) |
| B15.000: Hepatitis A, associated with liver coma |
| B16.000: Acute hepatitis B, associated with factors (co-infection), and associated with hepatic coma |
| B17.000: Acute factor (repeat) infection in hepatitis B virus carriers |
| B18.000: Chronic viral hepatitis B, associated with factors |
| B19.000: Viral hepatitis, associated with liver coma |
| Z22.500: The carrier of the viral hepatitis pathogen |
| Z22.500x002: carriers of hepatitis B viral pathogens |
| Z22.501: Hepatitis C virus carrier |
| Z22.502: hepatitis B surface antigen carrier |
| Z22.503: Hepatitis B, junior Yang |
| Z22.504: Hepatitis B small Sanyang |
| Z22.505: Positive for hepatitis E antibody |
| **2. Kidney disease** |
| N18.000: Chronic kidney failure, the others |
| N19.000: Kidney failure |
| D09.101 Renal carcinoma in situ |
| E 10. Type 2001 diabetes mellitus is associated with renal complications |
| N03.900x003 for chronic nephritis |
| N03.900x006 Chronic kidney disease |
| N03.900x007 for chronic glomerulopathy |
| N11.900 Chronic renal tubule-interstitial nephritis |
| N18.900x011 for chronic renal impairment |
| **3.Malignant tumor** |
| C00–C99 Malignant tumor |
| Z85.701 Personal history of hypotypic lymphoma |
| **4.Organ transplantation** |
| Z94.000 Renal transplant status |
| Z94.400 Liver transplant status |
| Z94.100 Heart transplant status |
| Z94.200 Lung transplant status |
| Z94.600 Bone transplant status |
| Z94.700 Corneal transplantation status |
| Z94.800x021 stem cell transplantation status |
| Z94.801 Intestinal transplant status |
| Z94.802 Bone marrow transplant status |
| Z94.900 Organ and tissue transplant status |
| **5.Other** |
| Z51.000: Course of radiation therapy |
| Z51.200 Chemistry therapy, the others |

| Supplementary Table 2. The identifying criteria for incident cancers. | | | |
| --- | --- | --- | --- |
| **ICD-10 codes** | **MAFLD** | **No MAFLD** | **Cancers** |
| C00-C99 | 291 | 290 | All cancers |
| C00-C10 | 4 | 2 | Oral cavity |
| C11-C13 | 9 | 12 | Pharynx |
| C15 | 2 | 9 | Esophagus |
| C16 | 14 | 23 | Stomach |
| C17-C21 | 49 | 55 | Duodenum, colon & rectum |
| C22 | 14 | 16 | Liver |
| C23-C24 | 5 | 3 | Biliary |
| C25 | 7 | 5 | Pancreas |
| C26 | 3 | 1 | Spleen |
| C32 | 5 | 1 | Laryngeal |
| C34 | 46 | 59 | Lung |
| C40 | 1 | 0 | Bone |
| C43 | 2 | 2 | Melanoma |
| C44 | 7 | 2 | Skin |
| C48 | 1 | 0 | Mesothelial |
| C49 | 0 | 1 | Soft tissues |
| C50 | 19 | 18 | Breast |
| C51-C57 | 21 | 17 | Labium, uterus, cervical & ovary |
| C61 | 18 | 17 | Prostate |
| C64-C65 | 9 | 4 | Renal pelvis |
| C66 | 2 | 1 | Ureter |
| C67 | 10 | 3 | Bladder |
| C68 | 2 | 1 | Urethra |
| C70-C72 | 7 | 3 | Brain & CNS |
| C73 | 22 | 19 | Thyroid |
| C74 | 1 | 0 | Adrenal cortex |
| C81 | 0 | 1 | Hodgkin lymphoma |
| C82-C85 | 7 | 13 | Non-Hodgkin lymphoma |
| C88 | 0 | 1 | Macroglobulinemia |
| C90 | 0 | 4 | Multiple myeloma |
| C91-C95 | 8 | 6 | Leukemia |
| C97 | 1 | 2 | Multiple sites of primary malignancy |

| Supplementary Table 3. Cancer incidence rates in subjects with and without MAFLD by gender. | | | | | | | | | | |
| --- | --- | --- | --- | --- | --- | --- | --- | --- | --- | --- |
| **Cancer site** | **Male** | | | | | **Female** | | | | |
|  | **Cancer incidence rates per 100,000 person-years** | | | | **P-value** | **Cancer incidence rates per 100,000 person-years** | | | | **P-value** |
|  | **All** | **MAFLD** | **No MAFLD** | **IRR(95%CI)** |  | **All** | **MAFLD** | **No MAFLD** | **IRR(95%CI)** |  |
| All cancers | 459.5 | 512.9 | 413.8 | 1.24 (0.99, 1.54) | 0.049 | 240.2 | 425.6 | 171 | 2.49 (1.92, 3.22) | <0.001 |
| Oral cavity | 5.5 | 8.9 | 2.5 | 3.50 (0.28, 183.92) | 0.247 | 2 | 3.6 | 1.3 | 2.68 (0.03, 210.31) | 0.468 |
| Pharynx | 23.2 | 23.7 | 22.8 | 1.04 (0.35, 3.03) | 0.939 | 3.9 | 3.6 | 4 | 0.89 (0.02, 11.12) | 0.922 |
| Esophagus | 9.6 | 5.9 | 12.7 | 0.47 (0.04, 2.85) | 0.351 | NA | NA | NA | NA | NA |
| Stomach | 32.8 | 20.8 | 43.2 | 0.48 (0.17, 1.22) | 0.096 | 12.7 | 25.2 | 8.1 | 3.13 (1.05, 9.30) | 0.041 |
| Duodenum, colon & rectum | 86.2 | 100.8 | 73.6 | 1.37 (0.81, 2.33) | 0.212 | 40.2 | 54.1 | 35 | 1.55 (0.76, 3.03) | 0.176 |
| Liver | 34.2 | 35.6 | 33 | 1.08 (0.45, 2.56) | 0.851 | 4.9 | 7.2 | 4 | 1.79 (0.15, 15.59) | 0.519 |
| Biliary | 5.5 | 5.9 | 5.1 | 1.17 (0.08, 16.11) | 0.877 | 3.9 | 10.8 | 1.3 | 8.04 (0.84, 77.275) | 0.071 |
| Pancreas | 13.7 | 14.8 | 12.7 | 1.17 (0.27, 5.07) | 0.806 | NA | NA | NA | NA | NA |
| Spleen | 4.1 | 5.9 | 2.5 | 2.34 (0.12, 137.79) | 0.476 | NA | NA | NA | NA | NA |
| Laryngeal | 8.2 | 14.8 | 2.5 | 5.84 (0.65, 276.17) | 0.068 | NA | NA | NA | NA | NA |
| Lung | 97.1 | 83 | 109.2 | 0.76 (0.45, 1.25) | 0.258 | 33.3 | 64.9 | 21.5 | 3.01 (1.54, 5.91) | 0.001 |
| Skin | 5.5 | 8.9 | 2.5 | 3.50 (0.28, 183.92) | 0.247 | 4.9 | 14.4 | 1.3 | 10.72 (1.20, 95.88) | 0.034 |
| Breast | NA | NA | NA | NA | NA | 35.3 | 68.5 | 22.9 | 2.99 (1.56, 5.76) | 0.001 |
| Labium, uterus, cervical & ovary | NA | NA | NA | NA | NA | 37.3 | 75.7 | 22.9 | 3.31 (1.75, 6.27) | <0.001 |
| Prostate | 47.9 | 53.4 | 43.2 | 1.24 (0.60, 2.55) | 0.53 | NA | NA | NA | NA | NA |
| Renal pelvis | 13.7 | 20.8 | 7.6 | 2.72 (0.62, 16.33) | 0.13 | 2.9 | 7.2 | 1.3 | 5.36 (0.28, 316.13) | 0.124 |
| Bladder | 15 | 23.7 | 7.6 | 3.11 (0.75, 18.23) | 0.077 | NA | NA | NA | NA | NA |
| Brain & CNS | 9.6 | 14.8 | 5.1 | 2.92 (0.48, 30.66) | 0.179 | 2.9 | 7.2 | 1.3 | 5.36 (0.28, 316.13) | 0.124 |
| Thyroid | 17.8 | 29.6 | 7.6 | 3.89 (1.07, 14.14) | 0.039 | 27.4 | 43.3 | 21.5 | 2.01 (0.87, 4.53) | 0.062 |
| Non-Hodgkin lymphoma | 16.4 | 14.8 | 17.8 | 0.83 (0.21, 3.05) | 0.757 | 7.8 | 7.2 | 8.1 | 0.89 (0.09, 4.99) | 0.89 |
| Leukemia | 10.9 | 11.9 | 10.2 | 1.17 (0.22, 6.27) | 0.826 | 6.9 | 14.4 | 4 | 3.57 (0.60, 24.39) | 0.075 |
| Males: follow-up duration, 73,124person-years, no MAFLD group: 39,392 person-years, MAFLD group; 33,732 person-years | | | | | | | | | | |
| Females: follow-up duration, 102,013 person-years; no MAFLD group: 74,286 person-years, MAFLD group; 27,727 person-years | | | | | | | | | | |
| CI, confidence interval; IRR, incidence rate ratio; MAFLD, metabolic dysfunction-associated fatty liver disease. | | | | | | | | | | |
| Incidence rate of cancer was calculated among females. | | | | | | | | | | |
| Incidence rate of cancer was calculated among males. | | | | | | | | | | |

| Supplementary Table 4. Association between MAFLD and development of cancers by gender. | | | | | | | | |
| --- | --- | --- | --- | --- | --- | --- | --- | --- |
| **Cancer site** | **Male** | | | | **Female** | | | |
|  | **Univariate analysis** | | **Multivariable analysis** | | **Univariate analysis** | | **Multivariable analysis** | |
|  | **HR (95% CI)** | **P-value** | **HR (95% CI)** | **P-value** | **HR (95% CI)** | **P-value** | **HR (95% CI)** | **P-value** |
| All cancers | 1.22 (0.99, 1.52） | 0.065 | 1.29 (1.04, 1.60） | 0.022 | 2.37 (1.84, 3.05） | <0.0001 | 1.42 (1.09, 1.85） | 0.01 |
| Oral | 3.41 (0.35, 32.76） | 0.288 | 3.02 (0.31, 29.56） | 0.343 | 2.85 (0.18, 45.49） | 0.46 | 2.51 (0.11, 56.38） | 0.563 |
| Pharynx | 1.02 (0.39, 2.65） | 0.963 | 1.06 (0.41, 2.75） | 0.91 | 0.81 (0.08, 7.74） | 0.851 | 0.46 (0.04, 4.76） | 0.516 |
| Esophagus | 0.46 (0.09, 2.38） | 0.357 | 0.49 (0.09, 2.55） | 0.398 | NA | NA | NA | NA |
| Stomach | 0.47 (0.20, 1.14） | 0.096 | 0.47 (0.19, 1.14） | 0.093 | 2.86 (0.96, 8.52） | 0.059 | 1.75 (0.55, 5.53） | 0.342 |
| Duodenum, colon & rectum | 1.36 (0.83, 2.22） | 0.229 | 1.46 (0.88, 2.40） | 0.141 | 1.38 (0.72, 2.64） | 0.333 | 0.65 (0.34, 1.26） | 0.202 |
| Liver | 1.06 (0.49, 2.33） | 0.879 | 1.09 (0.50, 2.40） | 0.828 | 1.54 (0.26, 9.25） | 0.636 | 0.75 (0.12, 4.66） | 0.759 |
| Pancreas | 1.16 (0.33, 3.99） | 0.819 | 1.22 (0.35, 4.23） | 0.757 | NA | NA | NA | NA |
| Spleen | 2.35 (0.21, 25.90） | 0.486 | 2.62 (0.23, 29.38） | 0.435 | NA | NA | NA | NA |
| Laryngeal | 5.74 (0.67, 49.10） | 0.111 | 5.03 (0.58, 43.79） | 0.143 | NA | NA | NA | NA |
| Lung | 0.75 (0.46, 1.20） | 0.231 | 0.80 (0.49, 1.29） | 0.364 | 2.87 (1.46, 5.62） | 0.002 | 1.36 (0.68, 2.73） | 0.38 |
| Skin | 3.49 (0.36, 33.56） | 0.279 | 4.32 (0.45, 41.73） | 0.207 | 10.05 (1.12, 89.93） | 0.039 | 4.40 (0.48, 39.89） | 0.188 |
| Breast | NA | NA | NA | NA | 2.90 (1.51, 5.59） | 0.001 | 1.96 (0.96, 3.98） | 0.064 |
| Labium, uterus, cervical & ovary | NA | NA | NA | NA | 2.97 (1.56, 5.68） | 0.001 | 2.24 (1.09, 4.60） | 0.029 |
| Prostate | 1.22 (0.63, 2.37） | 0.553 | 1.46 (0.75, 2.85） | 0.263 | NA | NA | NA | NA |
| Renal pelvis | 2.71 (0.70, 10.49） | 0.148 | 2.69 (0.69, 10.47） | 0.153 | 5.42 (0.49, 59.85） | 0.168 | 2.20 (0.20, 24.41） | 0.521 |
| Bladder | 3.06 (0.81, 11.54） | 0.098 | 3.31 (0.87, 12.56） | 0.079 | NA | NA | NA | NA |
| Brain & CNS | 2.89 (0.56, 14.90） | 0.205 | 3.20 (0.61, 16.64） | 0.167 | 5.45 (0.49, 60.23） | 0.167 | 2.80 (0.23, 34.44） | 0.422 |
| Thyroid | 3.84 (1.06, 13.95） | 0.041 | 4.42 (1.21, 16.22） | 0.025 | 1.94 (0.92, 4.11） | 0.082 | 3.37 (1.41, 8.04） | 0.006 |
| Non-Hodgkin lymphoma | 0.83 (0.26, 2.60） | 0.745 | 0.90 (0.29, 2.86） | 0.863 | 0.84 (0.17, 4.17） | 0.832 | 0.42 (0.08, 2.13） | 0.294 |
| Leukemia | 1.52 (0.34, 6.78） | 0.586 | 1.54 (0.34, 6.97） | 0.572 | 3.45 (0.77, 15.41） | 0.106 | 2.38 (0.47, 12.11） | 0.297 |
| The hazard ratios and p values represent the MAFLD group compared to the non-MAFLD group using a Cox proportional hazards regression model. | | | | | | | | |
| Multivariable analyses were adjusted for age, smoking status, alcohol status. | | | | | | | | |
| CI, confidence interval; HR, hazard ratio; MAFLD, metabolic dysfunction-associated fatty liver disease | | | | | | | | |
